# Dynamic resolution of functionally related gene sets in response to acute heat stress

**DOI:** 10.1186/1471-2199-8-46

**Published:** 2007-06-05

**Authors:** Joseph D Szustakowski, Penelope A Kosinski, Christine A Marrese, Jee-Hyung Lee, Stephen J Elliman, Nanguneri Nirmala, Daniel M Kemp

**Affiliations:** 1 Developmental and Molecular Pathways, Novartis Institutes for BioMedical Research, Cambridge, Massachusetts, USA; 2 Diabetes & Metabolism Disease Area, Novartis Institutes for BioMedical Research, Cambridge, Massachusetts, USA

## Abstract

**Background:**

Using a gene clustering strategy we determined intracellular pathway relationships within skeletal myotubes in response to an acute heat stress stimuli. Following heat shock, the transcriptome was analyzed by microarray in a temporal fashion to characterize the dynamic relationship of signaling pathways.

**Results:**

Bioinformatics analyses exposed coordination of functionally-related gene sets, depicting mechanism-based responses to heat shock. Protein turnover-related pathways were significantly affected including protein folding, pre-mRNA processing, mRNA splicing, proteolysis and proteasome-related pathways. Many responses were transient, tending to normalize within 24 hours.

**Conclusion:**

In summary, we show that the transcriptional response to acute cell stress is largely transient and proteosome-centric.

## Background

Damaged skeletal muscle tissue retains the capacity to self-repair through activation, expansion and fusion of resident satellite, progenitor cells [1-3]. However, the signaling mechanisms required to trigger satellite cell activation remain unclear in this context. Emerging pathways involved in the injury-response profile of skeletal muscle include Notch [4], Akt/Foxo [5] and NFκB [6,7], which appear to play distinct roles in regeneration, hypertrophy and atrophy. Although these signaling pathways are well established, there remains a dearth of molecular detail, including the transcriptional consequence of atrophic stimuli, and this aspect forms the focus of the current study.

We hypothesized that subtle variance in gene expression may underlie significant functional events within the cell. Consistently, previous studies have addressed the transcriptional profile of recovering muscle tissue following acute injury [8]. However, such studies rely on the analysis of individual genes, which is limiting from both statistical and biological perspectives. Here, we performed a study with the tenet that mechanistic data may be more readily revealed in the context of pathway-related gene clustering [9,10]. We employed Gene Set Enrichment Analysis (GSEA), a bioinformatics approach conceived to exploit functional regulation at the pathway level. The study focused on the dynamic expression profile of acutely stressed skeletal muscle cells, and the resultant data revealed major transcriptional adjustments in a wide range of functional categories. These findings may underscore a coordinated pathway-centric response, and reveal rational strategies for target discovery in atrophic muscle diseases.

## Results

**Figure 1:**
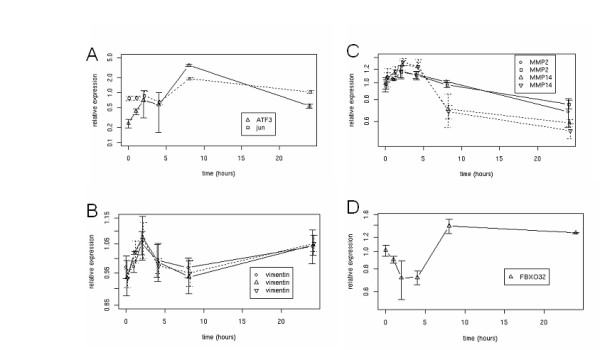
C2C12 cells recover from heat shock with a characteristic profile of injury response. Temporal gene expression profiles of heat shocked C2C12 myotubes (47°C for 30 minutes). Heat shock treatment was abated at 0 hours and cells were restored to growth conditions. mRNA was isolated at 1, 2, 4, 8 and 24 hours post-heat shock. (A-D) Transcriptional activity of the indicated genes was measured using Affymetrix GeneChips and data was analyzed using GeneSpring bioinformatics software. Data points represent the mean of three independent studies (± S.D). (E-G) C2C12 cells stably expressing Foxo3a-GFP were differentiated into myotubes (E), heat shocked and recovered for 3 hours (F) or treated with 20 μM LY294002 for 3 hours (G). Cells were then viewed under fluorescent light to observe localization of the protein.

In the present study, we exposed C2C12 skeletal myotubes to heat shock-induced damage in order to identify coordinate patterns of transcriptional regulation. To initially authenticate this cell-based injury model, we identified several stress-related biomarkers and regulatory patterns to confirm *bona fide *damage response in the cells. Following heat shock treatment, cells recovered in growth medium at 37°C, and gene expression profiles were generated at 0, 1, 2, 4, 8 and 24 hours post-treatment. ATF3 and c-jun are critically regulated in the neuronal response to damage and during degeneration. Furthermore, these genes may play an anti-apoptotic role in the survival response of neuronal cells [11-13]. Here, both ATF3 and c-jun transcript levels were upregulated in response to heat-shock, consistent with their reported coordinate role in cell damage response (Figure 1A). A transient peak in expression of both genes occurred at 8 hours and attenuated at 24 h post-damage induction, indicating a regulated damage-induced response. Vimentin, an intermediate filament protein that plays a role in wound healing [14] and response to thermal stress [15,16], was augmented immediately following heat shock, and peaked at 2 hours. This rapid response to cell stress was consistent with the role of vimentin in protection against cytotoxic effects (Figure 1B). Also, the membrane type 1-matrix metalloproteases MMP14 and MMP2, which are functional partners during skeletal development [17] and stress response in the context of vascular injury [18], were robustly co-regulated at the transcriptional level in response to heat shock of C2C12 skeletal myotubes. Their expression level rose within 2–4 hours of insult, followed by a repression of expression levels at 8 and 24 hours (Figure 1C).

Atrogin-1 is expressed specifically in skeletal and cardiac muscle, and has been shown to regulate muscle atrophy [19,20]. In response to heat shock, Atrogin-1 (*Fbxo32*) was upregulated after 8 hours, and this level of expression was maintained for at least 15 hours (Figure 1D). This dynamic profile is consistent with previous reports of muscle injury response, whereby the expression of atrogin-1 was concomitant with atrophy and recovery [21]. *Atrogin-1 *is regulated by Foxo transcription factors, via nuclear localization of Foxo in response to various stimuli that inhibit PI3K/Akt signaling and subsequently drive atrophy [5,22].

### Transient profiles of gene expression

**Figure 2:**
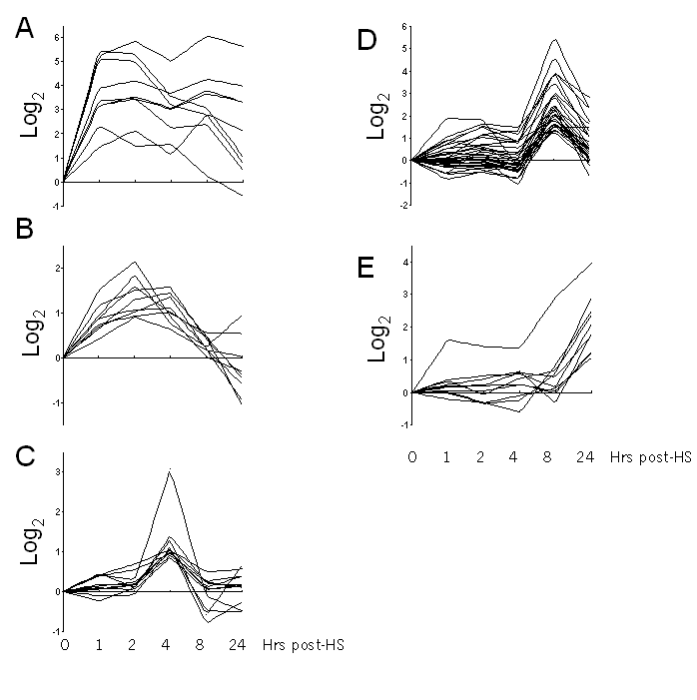
Temporal gene clusters during response to heat shock of C2C12 myotubes. Temporally upregulated genes were clustered by selection of probes that coregulated with a specific peak time between 1 and 24 hours post-heat shock, as determined from the normalized expression profile data. Expression profiles of each gene within clusters are represented for the entire 24 hour timecourse to reflect the transient nature of expression.

Of the approximately 46,000 probes arrayed on the gene chips, the most variable and dynamically regulated were characterized by basic clustering methodology (Figure 2). The most actively regulated group of genes immediately following treatment (ie. 1 hour post heat shock) unsurprisingly comprised a number of heat-shock proteins. *hsp1a*, *hsp1b *and *hsp8 *were among the most significantly upregulated transcripts at this time point (Figure 2A). Gene sets that clustered at 2, 4, 8 and 24 hours following heat shock are displayed in Figure 2 and are partially represented in Table 1. The 8 hour gene set was the largest group that displayed a significant upregulation, and is likely comprised of genes that were regulated downstream of immediate early genes. Therefore, this cluster may represent an important set of genes that functionally dictate the coordinated response of myogenic cells to stress or injury.

**Table 1:** Significantly upregulated genes at specific time points following heat shock

**1 Hours**	**8 Hours**
Hsp1A	Hsp105
Hsp1B	Hsp40 homolog – gi:12839599
Hsp8	Jun oncogene
Cystein rich protein 61	ATF3
	FBJ osteosarcoma oncogene
	
	Bcl2-associated athanogene 3
**2 Hours**	Developmentally and sexually retarded with transient immune abnormalities
	Endothelin 1
Procollagen, type VI, alpha 3	Placental growth factor
Procollagen, type V, alpha 1	Solute carrier family 19 (thiamine transproter), member 2
Procollagen, type I, alpha 1	AXIN1 up-regulated 1
Actin, beta, cytoplasmic	Moderate similarity to protein ref:NP_004410.2 (H.sapiens) phosphatase
Ribosomal protein S24	Heme oxygenase (decycling) 1
Low density lipoprotein receptor-related protein 1	Growth arrest and DNA-damage-inducible 45 gamma
Perlecan (heparan sulfate proteoglycan 2)	Myeloid differentiation primary response gene 116
Myosin heavy chain IX	v-maf musculoaponeurotic fibrosarcoma oncogene family, protein F (avian)
Early growth response 1	Dual specificity phosphatase 2
Expressed sequence – gi:2918507	Diphtheria toxin receptor
EST – gi:8772531	Transformed mouse 3T3 cell double minute 2
	Interferon-related developmental regulator 1
	
	Activity regulated cytoskeletal-associated protein
**4 Hours**	mRNA – gi:16877863
	DnaJ (Hsp40) homolog, subfamily A, member 4
Acidic (leucine-rich) nuclear phosphoprotein 32 family, member A	
	
Lysosomal acid lipase 1	
Lysyl oxidase-like 2	**24 Hours**
ATPase, Na+/K+ transporting, alpha 1 polypeptide	
Nidogen 1	Clusterin
Guanine nucleotide binding protein, alpha inhibiting 2	Monocyte to macrophage differentiation-associated
Protein tyrosine phosphatase 4a2	Purinergic receptor P2X, ligand-gated ion channel, 7
CD151 antigen	Histone 1, H4i
Similar to hypothetical protein MGC4368 – gi:15655075	Aldehyde dehydrogenase family 3, subfamily A1
Expressed sequence – gi:2519775	Pyridoxal (pyridoxine, vitamin B6) kinase
cDNA – gi:9515377	CDP-diacylglycerol synthase (phosphatidate cytidylyltransferase) 2
	Protein kinase C-like 1
	Related to uridine kinase – gi:12844097
	mRNA – gi:4779190

### Gene set enrichment analysis

**Figure 3:**
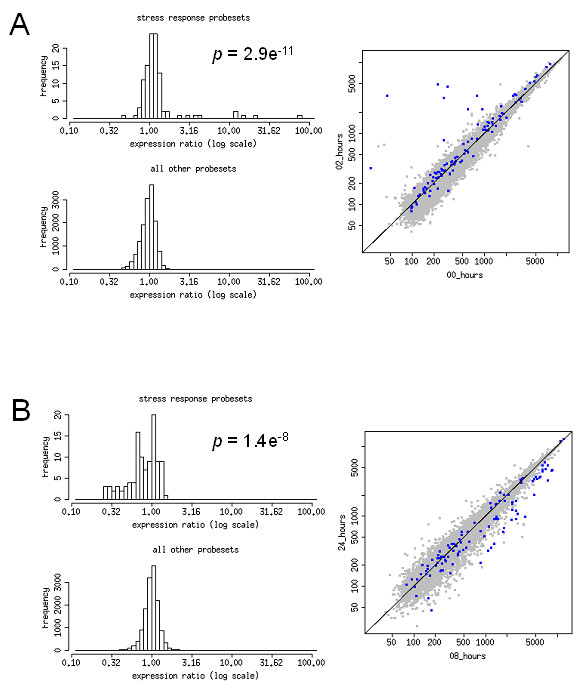
Stress response gene set is bi-directional during response to heat shock in C2C12 cell. Distribution in stress response-related gene sets during response to heat shock of C2C12 myotubes. All probes representing the stress response gene set are presented as an expression ratio of time *a *against time *b*. Frequency (*y-axis*) represents the number of probes in each gene set with the corresponding expression ratio (*x-axis*). The statistical significance of the mean expression variance ratio for the gene set relative to all other probesets is indicated. Variance between 0 – 8 hours (A) and 8 – 24 hours (B) post-heat shock are depicted in the figure. Dotplot representations of GSEA as a function of C2C12 response to heat shock is also presented. The *x-axis *of each plot represents expression levels at time *a*, and the *y-axis *represents expression at time *b*. Each dot represents a single probe on the GeneChip. The total probe set (grey dots) and the specific gene set (blue dots) are presented on the same plot to give direct perspective of gene set regulation. The stress response probe set is represented by 122 gene probes, previously annotated as related to the stress response. The list is attached in Additional file 1.

We employed a the gene set enrichment analysis strategy [10] to cluster functionally related genes in order to consider shifts in pathway activity. Individual gene sets were mined from various publicly and privately available data sources, and methodologies for the curation of functional pathways varied between sources. In all, a total of 789 gene sets made up the collection that was employed in this study, and the approach was employed to facilitate potential mechanistic insight to the myogenic cell response to injury [10]. As a control, we looked at the gene set for stress response at the 8 hour time point (Figure 3A). This probe set was significantly upregulated (p < 1.0e^-36^), relative to the basal expression distribution (Figure 3–3A). Furthermore, a subsidence in the stress response could be observed between 8 and 24 hours post-heat shock treatment as the gene set underwent a significant downregulation (p = 1.4e^-8^) that may reflect a normalization of the cell's condition (Figure 3B). These data clearly demonstrate the statistical strength of pathway analysis in the context of the heat shock response.

**Figure 4:**
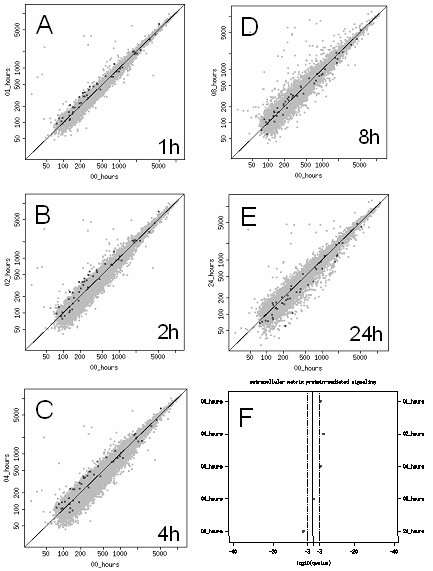
Dynamic regulation of the EMP-M gene set following heat shock. (A-E) Dotplot diagrams represent the temporal shift in mean expression levels of the EMP-M pathway gene set, relative to the 0 hour time point. (F), an overview of the dynamic pattern of mean expression levels of the EMP-M pathway gene set. Shifts to the right or left of the vertical center line represent statistical significance of directed shift (*q *value), rather than the mean expression level *per se*. The list of genes/probes that constitute the EMP-M pathway gene set can be found in Additional file 1.

To gain better insight into the dynamic nature of regulated functional pathways, we traced the expression pattern of specific gene sets within the time course, such as the extracellular matrix protein-mediated signaling (EMP-M) (Figure 4, see Additional file 1). The analysis revealed a pattern of regulation that reflected an initial increase in pathway activity at 1, 2, and 4 hours, followed by a time-dependent decrease in expression through 8 and 24 hours post treatment. Therefore, the overall response in the EMP-M pathway to heat shock reflected an augmentation immediately following treatment, followed by a subsidence and eventual decrease in functional pathway expression after 24 hours. The transient nature of the pathway response was clearly defined with this bioinformatics approach, and is presented in Figure 4F.

**Figure 5:**
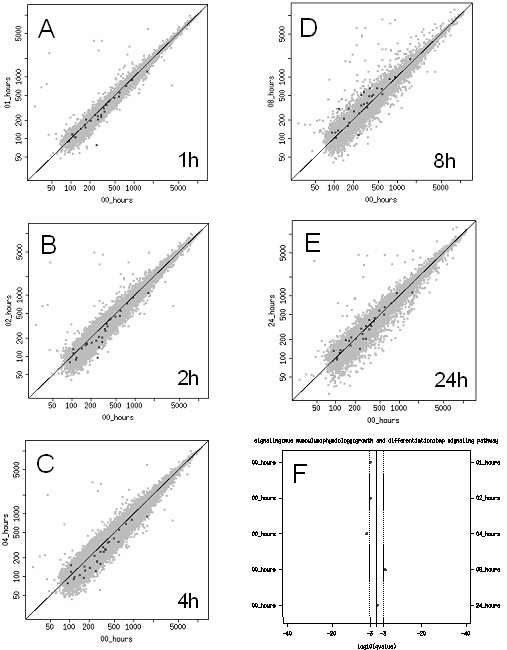
Dynamic regulation of the BMP gene set following heat shock. (A-E) Dotplot diagrams represent the temporal shift in mean expression levels of the BMP pathway gene set, relative to the 0 hour time point. (F), an overview of the dynamic pattern of mean expression levels of the BMP pathway gene set. Shifts to the right or left of the vertical center line represent statistical significance of directed shift (*q *value), rather than the mean expression level *per se*. The list of genes/probes that constitute the BMP pathway gene set can be found in Additional file 1.

A similar analysis was carried out on the BMP signaling pathway (see Additional file 1). When traced as a function of time over the 24 hour period, the BMP signaling pathway was initially decreased at 1, 2 and 4 hours at the transcriptional level, and recovered through 8 and 24 hours post treatment (Figure 5). This result was consistent with the established role of BMP signaling in antagonizing myogenic specification during development, and in regenerating adult muscle tissue [23]. Furthermore, along with the stress response pathway and the EMP-M pathway expression traces, it appears that much of the initial response to heat shock was reversed within 24 hours of recovery.

**Figure 6:**
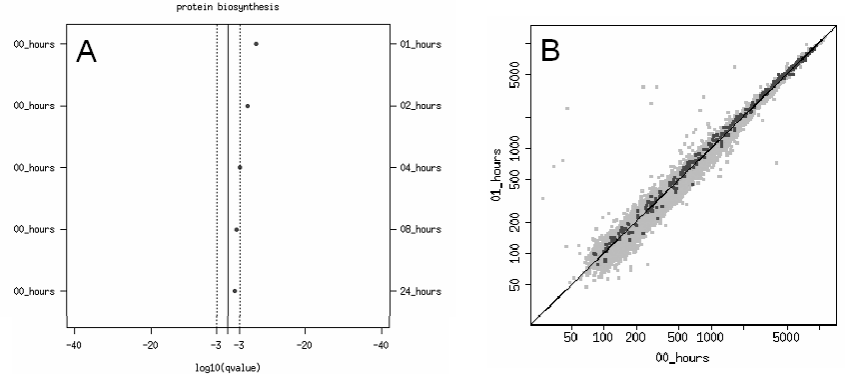
Dynamic response of the protein biosynthesis pathway following heat shock. (A) Overview depiction of the dynamic response of the protein biosynthesis pathway gene set following heat shock over 24 hours. Data is represented as for Figure 5. (B) Dotplot diagram showing the upregulation in expression of the same gene set after 1 hour post-heat shock.

Analysis of the most active gene sets through the entirety of the time-course revealed a notable phenomenon, typified by the dynamic profile of the protein biosynthesis pathway gene set. In response to heat shock treatment, the genes in this pathway underwent a significant and immediate upregulation, which gradually declined over the next 24 hours (Figure 6). The trend for this particular functional pathway suggested that protein turnover was highly regulated in the response to heat shock-induced stress, and this hypothesis was further supported by the apparent upregulation of several related pathways involved in the expression and degradation of proteins.

After 8 hours recovery from heat shock, a selection of gene sets related to core gene expression machinery and protein turnover, were significantly upregulated (Table 2). This general increase in protein metabolism pathways as an immediate response to injury may reflect the clearance and replacement (turnover) of the protein complement of the cell.

**Table 2:** Most significantly upregulated gene sets at 8 hours post heat shock

Functional Pathway	No. of probes in set	*p*-value	*q*-value
Protein metabolism and modification	1794	< 1e^-31^	< 1e^-31^
Protein folding	173	1.1e^-13^	1.8e^-11^
Nuclear transport	87	8.4e^-8^	7.9e^-6^
Pre-mRNA processing	209	4.0e^-7^	2.1e^-5^
Proteolysis	461	1.7e^-6^	6.2e^-5^
mRNA transcription regulation	676	9.1e^-7^	4.2e^-5^
Protein complex assembly	53	1.2e^-6^	4.8e^-5^
mRNA splicing	156	7.5e^-6^	2.0e^-4^
Proteasome	37	1.3e^-4^	1.9e^-3^

## Discussion and Conclusion

This study was conducted to explore changes in gene expression following acute injury of C2C12 myotubes, and to reveal regulation of pathways during initial recovery. 866 individual transcripts represented on the microarray varied more than 2 fold over the course of the study, and the data revealed significant transcriptional changes in a wide variety of functional classes following heat shock damage to the cells. We devised the current study with two distinct dimensions. Firstly, to trace the time-course of recovery over a 24 hour period, and secondly, to analyze in a pathway-centric manner in order to extract hypotheses of coordinated molecular mechanisms that occur during cellular stress and recovery. Two notable pathways significantly modulated during recovery were the BMP signaling pathway, and the extracellular matrix-protein mediated signaling pathway, both of which are known to function in myogenesis, either by directing lineage specification (BMP) or structural/regenerative signaling (ECM-M). Additionally, the muscle-specific E3 ligase gene, *Atrogin-1 *was upregulated significantly, consistent with its reported role in mediating muscle atrophy in response to catabolic signals [19], and this in turn was coincident with our observation of nuclear translocation of Foxo3a following heat shock [5,22]. This reconstruction of a well documented atrophic pathway served to validate the heat shock treatment paradigm as a useful model of acute muscle stress.

The experimental approach carried out in this study demonstrated the value of characterizing cellular responses in a temporal fashion, as transcriptional regulation was commonly found to be non-static between contiguous time points within the first 24 hours of treatment, and often presented transient regulation that required a time-course dimension to clarify.

A notable trend that emerged from the data was an apparent increase in protein turnover apparatus-related gene sets. The most significantly upregulated gene sets included those for both transcriptional activity, translational activity and protein modification, as well as the proteolysis and proteosome pathways. This suggests that a primary response of skeletal myotubes to heat shock treatment is to renew the protein complement of the cell, presumably as a defense mechanism against proteomic damage, and potential aberrant consequences in the function or integrity of the cell. The data also show that much of this response is transient, and appears to normalize within 24 hours of the acute insult at the transcriptional level.

In summary, the data presented in this report demonstrate a dynamic resolution of gene expression in response to acute stress that implicates waves of pathway activity in the cell's metabolic recovery. Further validation of these data are required, however, in order to demonstrate clearly the practical nature of these pathways as part of the functional stress response profile.

## Methods

### Cell culture and treatment

C2C12 mouse skeletal myoblasts were cultured in DMEM high glucose with 10% FBS and 1% penicillin/streptomycin (Gibco), and maintained at 37°C and 5% CO_2_. Cells were grown to confluence and then differentiated for seven days in media containing 3% fetal bovine serum. Cells were heat shocked at 47°C for 30 minutes, and returned to the incubator at 37°C. After heat shock, cells were harvested at 1, 2, 4, 8, and 24 hours and processed to isolate the RNA using the RNeasy kit (Qiagen). Myotubes were isolated for expression profiling by serially exposing the culture to increasing concentrations of trypsin. This allowed for the multinucleated myotubes to become detached from the plate, while the mononucleate myoblasts remained adherent. This process ensured that we were profiling differentiated skeletal myotubes only, without contamination of myoblasts. 20 μg RNA per sample was microarrayed using the Mouse Affymetrix MOE430 Plus 2.0 chip. The heat-shock treatment conditions were determined not to be cytotoxic, as continued culture in normal growth conditions was sufficient for full recovery of the cells (data not shown).

### Bioinformatics

Raw intensity files were imported into GeneSpring^® ^v.6.2 (Silicon Genetics, US) and filtered such that genes with a raw expression level of less than 100 were excluded from any future analyses. The resulting gene list was filtered to identify genes with a fold change increase or decrease of 2 or greater. Statistically significant genes were found using one-way ANOVA with time as variable and no multiple testing correction.

An in-house implementation of the Gene Set Enrichment Analysis method [10] was used to analyze microarray data. As input, GSEA requires microarray data from two conditions to be compared (*e.g*. myoblasts vs myotubes). Genes with expression levels below 100 on more than 75% of the Genechips were discarded. Then, the relative expression level under *condition*_1 _and *condition*_2 _was computed as an expression ratio *r*_*i*_

ri=μi,1μi,2
 MathType@MTEF@5@5@+=feaafiart1ev1aaatCvAUfKttLearuWrP9MDH5MBPbIqV92AaeXatLxBI9gBaebbnrfifHhDYfgasaacH8akY=wiFfYdH8Gipec8Eeeu0xXdbba9frFj0=OqFfea0dXdd9vqai=hGuQ8kuc9pgc9s8qqaq=dirpe0xb9q8qiLsFr0=vr0=vr0dc8meaabaqaciaacaGaaeqabaqabeGadaaakeaacqWGYbGCdaWgaaWcbaGaemyAaKgabeaakiabg2da9maalaaabaacciGae8hVd02aaSbaaSqaaiabdMgaPjabcYcaSiabigdaXaqabaaakeaacqWF8oqBdaWgaaWcbaGaemyAaKMaeiilaWIaeGOmaidabeaaaaaaaa@3AE8@

where *μ*_*i*,*j *_is the average expression value for gene *i *under *condition*_*j*_. Genes were then sorted according to their expression ratios. Next, the collection of available gene sets were projected onto the sorted gene list. In essence, this step applies *a priori *biological knowledge to the experimental data to identify functionally related genes that are expressed in a coordinated fashion. Gene sets were processed individually and used to partition the genes into two groups: those gene in a pathway, and all other genes measured on the Genechip. A two-tailed Wilcoxon rank-sum test was then calculated to determine if the genes in each gene set were enriched at either the top or bottom of the sorted list. The Wilcoxon p-values were then transformed into false discovery rate q-values [24,25] as a means of multiple hypothesis testing correction.

## Authors' contributions

JDS developed the bioinformatics algorithms and analysis. PAK designed and performed the heat shock experiment. CAM performed statistical analysis. JHL and SJE participated in the design and coordination of the study. NRN and DMK conceived of the study and drafted the manuscript. All authors read and approved the final manuscript.

## Supplementary Material

Additional File 1 Heat Shock supplementary probe lists. Table of genes that form the extracellular matrix protein-mediated signaling probe set, and the heat shock probe set, respectively. Click here for file
